# Supplementary material for: Lipid droplets and ferritin heavy chain: a devilish liaison in human cancer cell radioresistance
Source: eLife. 2021 Sep 9;10:e72943. doi: 10.7554/eLife.72943 (PMC8497056; doi:10.7554/eLife.72943)
Supplement: Figure 4—source data 1. [file elife-72943-fig4-data1.pptx]

## Slide 1
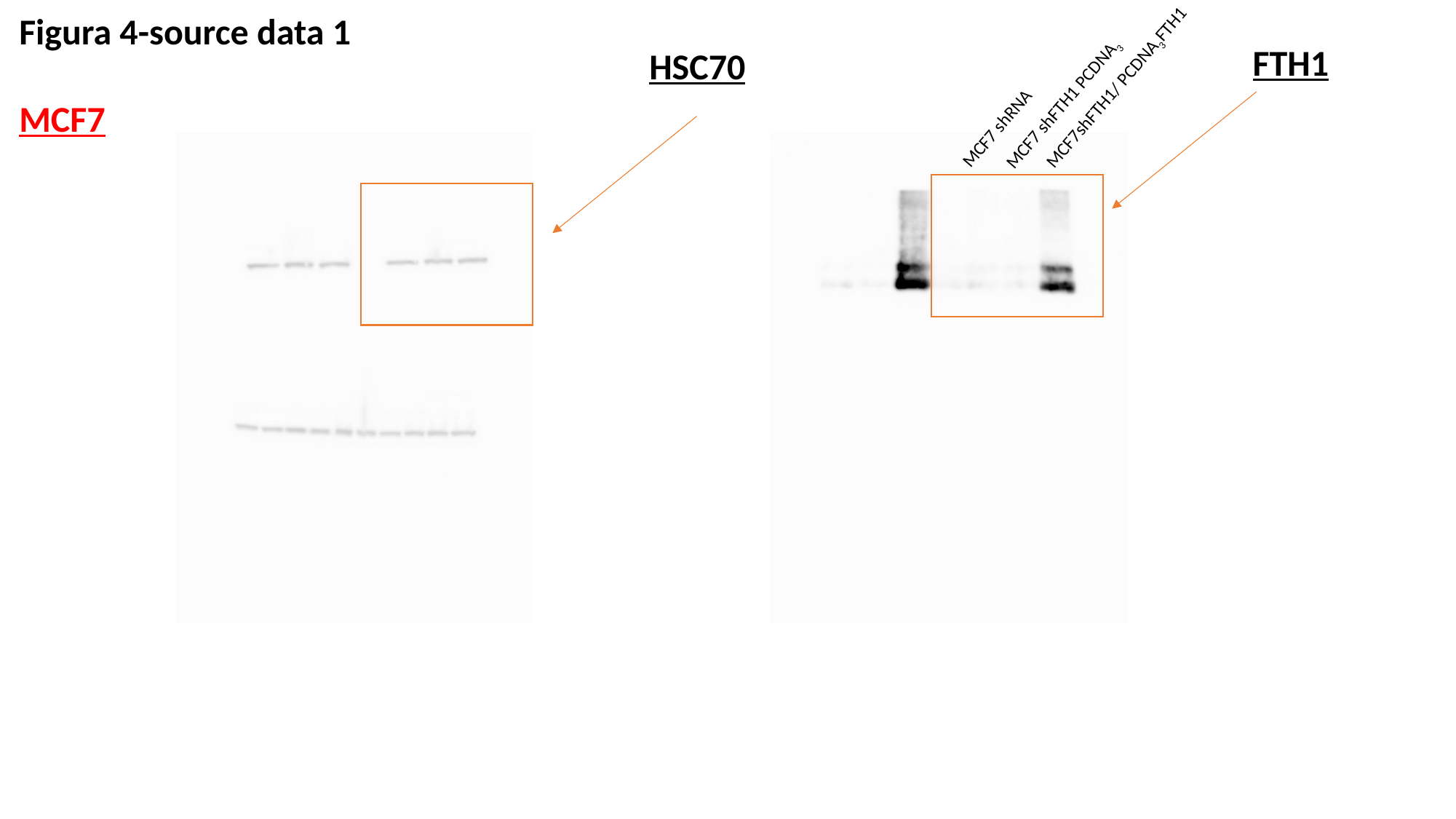

Figura 4-source data 1
MCF7
FTH1
HSC70
MCF7shFTH1/ PCDNA3FTH1
MCF7 shFTH1 PCDNA3
MCF7 shRNA

## Slide 2
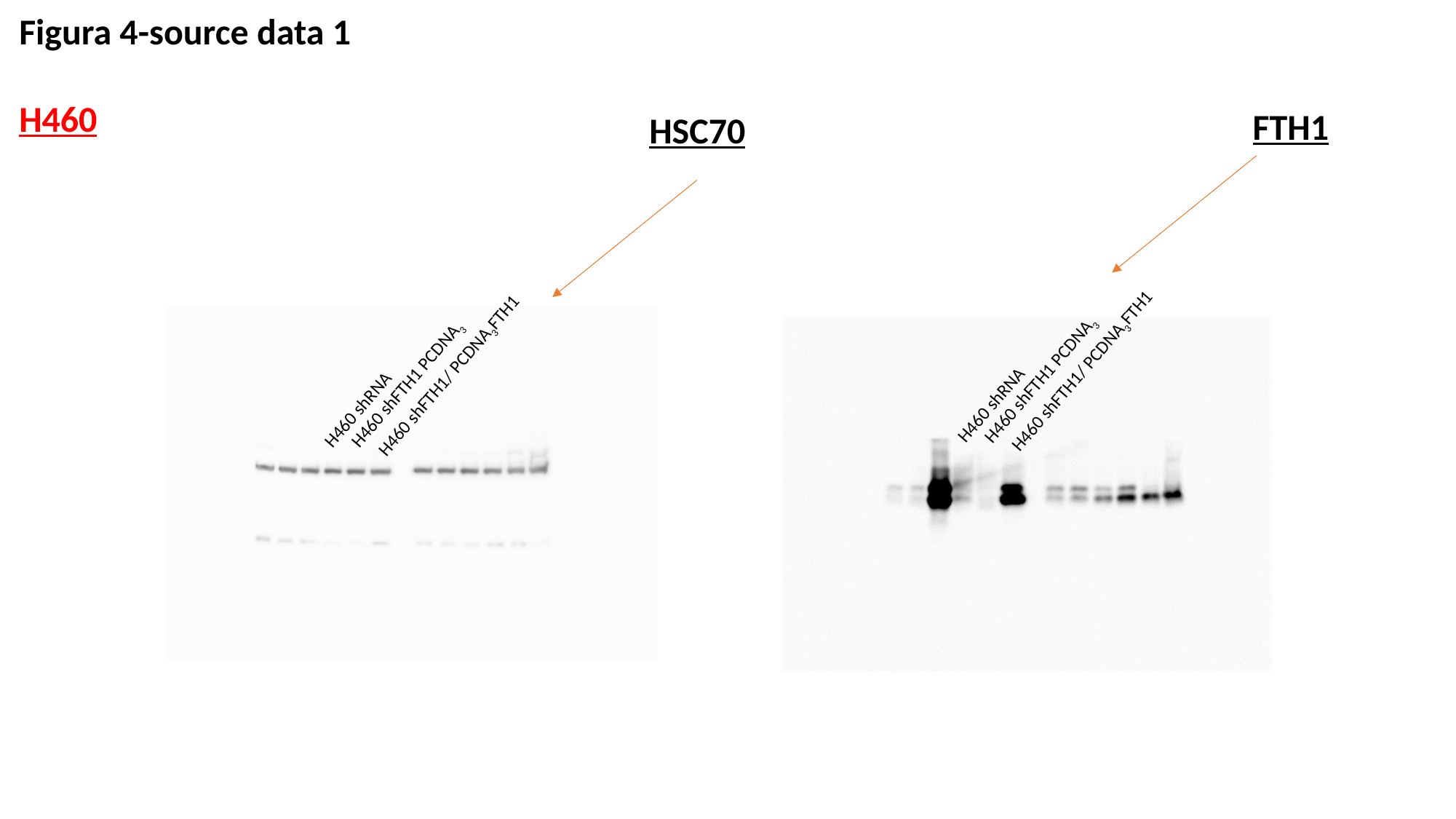

Figura 4-source data 1
H460
FTH1
HSC70
H460 shFTH1/ PCDNA3FTH1
H460 shFTH1/ PCDNA3FTH1
H460 shFTH1 PCDNA3
H460 shFTH1 PCDNA3
H460 shRNA
H460 shRNA
