# Supplementary figures and images for: Lipid droplets and ferritin heavy chain: a devilish liaison in human cancer cell radioresistance
Source: eLife. 2021 Sep 9;10:e72943. doi: 10.7554/eLife.72943 (PMC8497056; doi:10.7554/eLife.72943)

## Slide 1
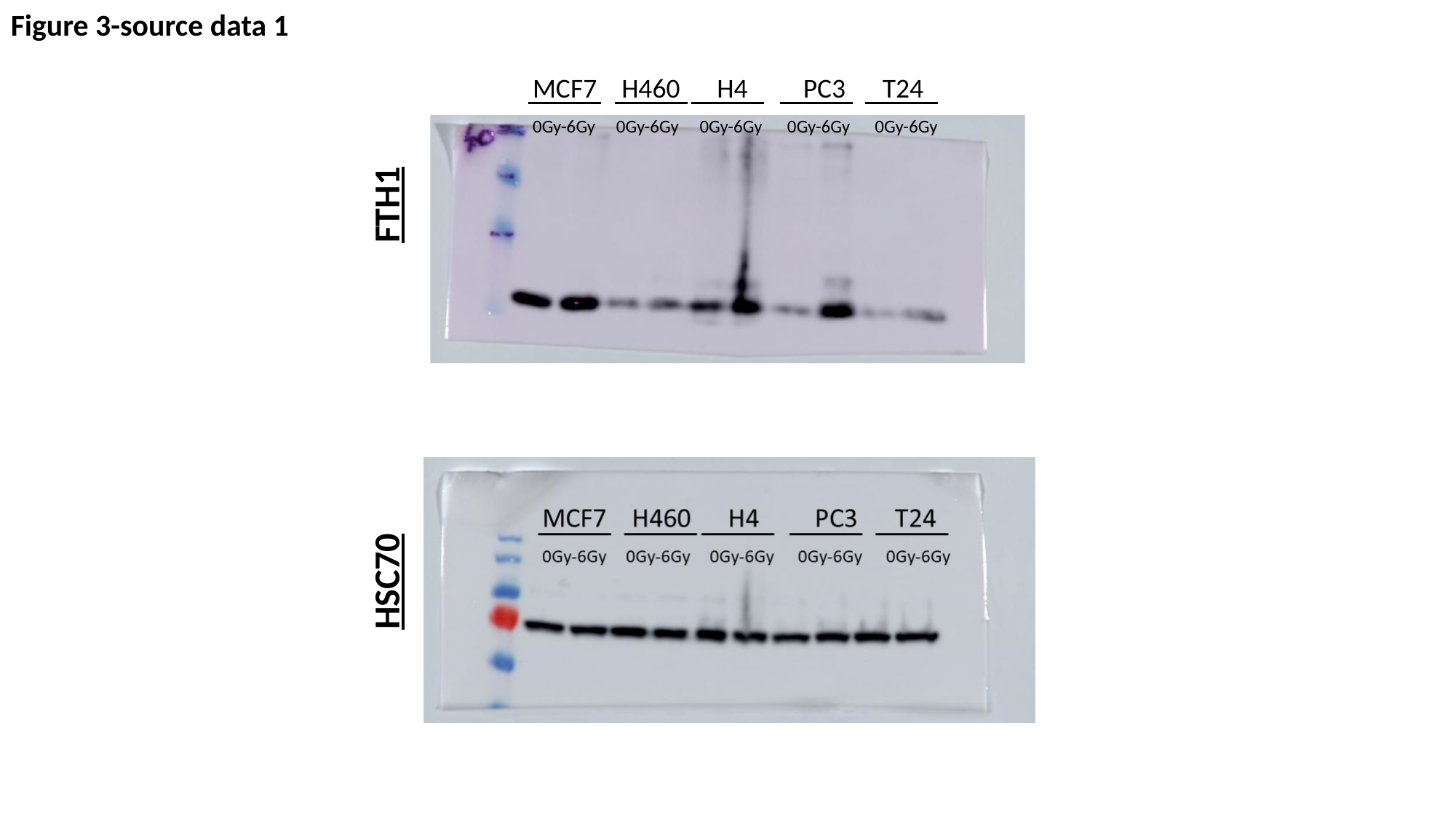

Figure 3-source data 1
MCF7 H460 H4 PC3 T24
0Gy-6Gy 0Gy-6Gy 0Gy-6Gy 0Gy-6Gy 0Gy-6Gy
FTH1
HSC70

Supplement: Source data 1. [file elife-72943-supp1.zip › Source Data/Figure 3-source data 1.pptx]
